# The role of metagenomic next-generation sequencing in diagnosing and managing post-kidney transplantation infections

**DOI:** 10.3389/fcimb.2024.1473068

**Published:** 2025-01-07

**Authors:** Hao Wu, Hongliang Cao, Xin Gao, Chengdong Shi, Lei Wang, Baoshan Gao

**Affiliations:** Department of Urology II, The First Hospital of Jilin University, Changchun, China

**Keywords:** kidney transplantation, metagenomic next-generation sequencing, infection, pathogens, diagnosis

## Abstract

Kidney transplantation (KT) is a life-saving treatment for patients with end-stage renal disease, but post-transplant infections remain one of the most significant challenges. These infections, caused by a variety of pathogens, can lead to prolonged hospitalization, graft dysfunction, and even mortality, particularly in immunocompromised patients. Traditional diagnostic methods often fail to identify the causative organisms in a timely manner, leading to delays in treatment and poorer patient outcomes. This review explores the application of metagenomic next-generation sequencing (mNGS) in the diagnosis of post-KT infections. mNGS allows for the rapid, comprehensive detection of a wide range of pathogens, including bacteria, viruses, fungi, and parasites, without the need for culture-based techniques. We discuss the advantages of mNGS in early and accurate pathogen identification, its role in improving patient management, and the potential challenges in its clinical implementation. Additionally, we consider the future prospects of mNGS in overcoming current diagnostic limitations and its potential for guiding targeted therapies, particularly in detecting antimicrobial resistance and emerging pathogens. This review emphasizes the promise of mNGS as an essential tool in improving the diagnosis and treatment of infections in KT recipients.

## 1 Introduction

Kidney transplantation (KT) is a surgical procedure that replaces a failed kidney with a functioning one from a donor, offering a life-saving treatment for patients with end-stage renal disease (ESRD) (Reimold et al., 2024). This procedure is especially critical for individuals whose renal function cannot be adequately supported by dialysis or other medical management (Navarrete, 2009; Unruh and Dew, 2014; Viklicky et al., 2020). Over the years, advancements in surgical techniques and immunosuppressive therapies have significantly improved the success rate and accessibility of KT (Lim et al., 2017; Szumilas et al., 2023). However, long-term postoperative management remains a major challenge, with complications such as graft rejection and infection posing significant risks to patient recovery and long-term outcomes (Kim et al., 2023; Voora et al., 2023).

Post-transplant infections are among the most common and severe complications following KT (Agrawal et al., 2022). These infections, caused by bacteria, viruses, fungi, or parasites, are a major clinical concern due to the lifelong use of immunosuppressive therapy, which compromises the immune system’s ability to fight off pathogens (Illesy et al., 2016; Dandamudi et al., 2019; Møller et al., 2021). Such infections can result in prolonged hospitalization, impaired graft function, and in severe cases, mortality. Early and accurate diagnosis of these infections is critical to ensuring timely intervention and effective treatment (Bharati et al., 2023; Pajenda et al., 2023; Grasberger et al., 2024). The selection of appropriate diagnostic methods is a key factor in identifying causative pathogens and guiding targeted therapy, thus improving outcomes for KT recipients (Bharati et al., 2023).

This review aims to summarize the currently available diagnostic methods for post-transplant infections in KT recipients, with a focus on the application of metagenomic next-generation sequencing (mNGS). We highlight the utility of mNGS in diagnosing various types of post-transplant infections, such as pulmonary, urinary tract, and bloodstream infections. Furthermore, we explore the current applications of mNGS in identifying specific pathogens associated with these infections. Lastly, we discuss the limitations and challenges of using mNGS in clinical practice and provide insights into potential improvements that could enhance its application in the management of post-transplant infections, thereby offering valuable guidance for its future use.

## 2 Diagnostic methods for post-transplant infections in KT

Post-transplant infections are among the most critical complications following KT, particularly in cases of complex infections involving polymicrobial or drug-resistant pathogens (Fishman, 2017; Agrawal et al., 2022). Early and accurate identification of causative pathogens is essential for effective treatment, graft survival, and preventing severe complications (Cippà et al., 2015). Conventional methods for pathogen detection, which remain widely utilized in clinical practice, include microbial culture, microscopy, serological tests, molecular diagnostic techniques, and mass spectrometry analysis (McAteer and Tamma, 2024). Microbial culture, often considered the gold standard, involves inoculating clinical specimens onto selective or differential media to promote the growth of specific microorganisms. It enables the isolation of viable organisms and facilitates antimicrobial susceptibility testing, making it a cornerstone of clinical microbiology. However, its utility is limited in time-sensitive scenarios due to the prolonged incubation period required for certain pathogens and its inability to detect fastidious or non-culturable organisms (Kim et al., 2020; Kobayashi et al., 2021). Microscopy, based on direct visualization of pathogens in stained clinical samples, offers rapid preliminary information and is particularly useful for identifying morphologically distinct pathogens. Despite its simplicity, microscopy often lacks sensitivity and specificity, especially when pathogen loads are low (Lunn et al., 2010; Ernstsen et al., 2017; Laketa, 2018). Molecular diagnostic techniques, such as polymerase chain reaction (PCR), have revolutionized infectious disease diagnostics by enabling the rapid and highly specific detection of pathogens based on their nucleic acid sequences. While PCR-based methods are powerful tools, they are inherently limited by their dependence on prior knowledge of the target sequence, making them less effective for detecting unexpected or unknown pathogens (Tsalik et al., 2018; Liu et al., 2023; Chen et al., 2024). Serological tests, such as enzyme-linked immunosorbent assays (ELISA), are commonly used for detecting pathogen-specific antigens or host antibodies, particularly in viral infections. These tests provide rapid and reliable results but may struggle to distinguish active infections from past exposures, complicating interpretation in certain clinical contexts (Lapošová et al., 2016; Luo et al., 2022; Chen et al., 2023). Mass spectrometry analysis, particularly matrix-assisted laser desorption/ionization time-of-flight mass spectrometry (MALDI-TOF MS), has emerged as a valuable tool in clinical microbiology for the rapid identification of microorganisms. This technique ionizes microbial proteins to generate mass spectra, which are then compared against reference databases to identify specific pathogens. MALDI-TOF MS offers high-throughput capabilities and rapid turnaround times, significantly enhancing the speed of pathogen identification. However, its effectiveness depends on the quality and comprehensiveness of the reference databases, and it may not reliably identify novel or rare pathogens (Lamy et al., 2020; Ohyama et al., 2020; Kondori et al., 2021). Collectively, these conventional methods have significantly advanced the diagnosis of post-transplant infections, but their limitations underscore the need for innovative diagnostic approaches (**Table 1**).

**Table 1:** Comparison of pathogen detection methods.

Method	Detection Scope	Sensitivity	Specificity	Time Required	Cost	Application Scenarios
Culture	Specific (requires appropriate medium)	Moderate	High (with susceptibility testing)	Long (days to weeks)	Low	Routine diagnosis of bacteria and some fungi
Microscopy	Limited (specific morphology)	Low	Low	Short (immediate)	Low	Simple detection, e.g., malaria, parasites
PCR	Specific (requires known target sequence)	High	High	Short (hours)	Moderate	Rapid detection of specific pathogens
ELISA/ Immunology	Limited (antigen-antibody related)	Moderate	Moderate (cross-reactivity possible)	Short (hours)	Low to moderate	Screening for viral antigens and antibodies
Mass Spectrometry	Known pathogen protein databases	High	High	Short (hours)	Moderate	Rapid identification of microorganisms
mNGS	Broad (known and unknown pathogens)	Very high	Moderate	Moderate	High	Difficult infections, complex infections, and emerging pathogens

mNGS, Metagenomic next-generation sequencing, PCR, polymerase chain reaction, ELISA, enzyme-linked immunosorbent assays.

**Figure 1:**
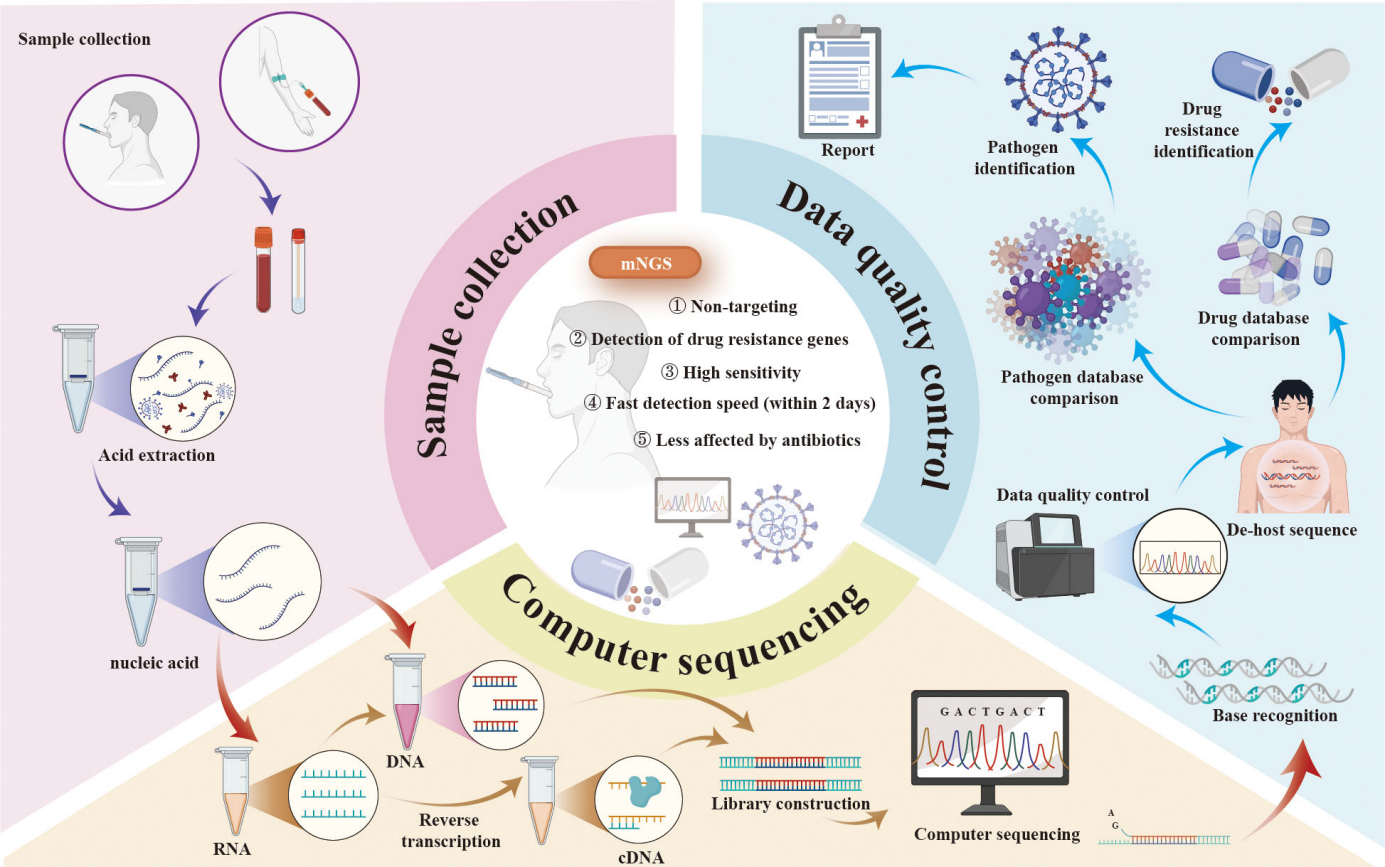
The protocol, advantages, and applications of mNGS. mNGS, metagenomic next-generation sequencing.

mNGS represents a transformative advance in pathogen detection, offering an unbiased and comprehensive approach that is particularly suited to the complex infections often encountered in KT recipients (Hao et al., 2023). Unlike conventional methods, mNGS does not rely on predefined assumptions about the causative pathogen. Instead, it sequences all nucleic acids (DNA or RNA) present in a clinical sample, enabling the simultaneous detection of bacteria, viruses, fungi, and parasites (Han et al., 2019). The typical workflow of mNGS begins with sample preparation, where nucleic acids are extracted from the specimen, and host DNA or RNA is depleted to enhance the detection of microbial sequences. High-throughput sequencing is then performed using advanced platforms such as Illumina or Oxford Nanopore, generating massive volumes of data that are subsequently analyzed using bioinformatics tools to align sequences with reference databases, identify pathogens, and exclude contaminants or background noise (Zhong et al., 2021; Bloemen et al., 2023; Cai et al., 2023; Ying et al., 2024) (**Figure 1**). The results are interpreted in conjunction with the clinical context to distinguish true pathogens from non-pathogenic or environmental organisms (Cai et al., 2023).

mNGS has undergone significant advancements, transitioning from Sanger sequencing to second- and third-generation technologies (Goodwin et al., 2016; Ghosh et al., 2018; Hu et al., 2021). Short-read platforms, such as Illumina, revolutionized genomic research in the mid-2000s by significantly increasing throughput and reducing costs, while long-read platforms like PacBio and Oxford Nanopore addressed limitations of short reads, including difficulties in resolving structural variants and repetitive regions (Goodwin et al., 2016; Ghosh et al., 2018; Hu et al., 2021). These innovations have expanded the applications of mNGS in areas such as diagnostics, pathogen detection, and environmental metagenomics.

Compared to traditional methods such as culture and PCR assays, mNGS offers higher sensitivity and faster detection, often delivering results within 24 hours (Chen et al., 2023; Wang et al., 2023). This rapid turnaround enables timely and targeted antimicrobial therapy, improving treatment efficacy and reducing the adverse consequences of antibiotic misuse. By enhancing the accuracy of pathogen identification, mNGS helps shorten the time to appropriate treatment, lowers healthcare costs, and significantly improves patient outcomes (Rodino and Simner, 2024). Additionally, mNGS provides valuable insights into pathogen resistance, virulence factors, and genomic variations, making it a cornerstone of precision medicine. Studies have highlighted its effectiveness in diagnosing mixed infections, detecting rare or low-frequency pathogens, and assessing host immune responses (Sun et al., 2022; Zhao et al., 2022; Zheng et al., 2022). A notable example of mNGS's impact is its role in the rapid identification of SARS-CoV-2, which expedited the development of targeted treatments and vaccines for COVID-19 (Zhong et al., 2021). In summary, mNGS represents a powerful, comprehensive diagnostic approach, revolutionizing the management of infections through enhanced accuracy, reduced treatment delays, and informed use of antimicrobials. Its contributions to precision medicine and public health underscore its critical role in modern infectious disease diagnostics (**Figure 1**).

## 3 Applications of mNGS in post-KT infections

Recent studies have explored the application of mNGS in diagnosing post-transplant infections in KT recipients, highlighting its potential to overcome many of the limitations associated with traditional diagnostics. By enabling early and precise pathogen identification, mNGS has demonstrated significant clinical value in guiding targeted therapies, improving patient outcomes, and optimizing infection management in this vulnerable population. As research continues to refine this technology, mNGS holds promise for transforming the diagnosis and treatment of post-transplant infections, offering a powerful tool for addressing the complex challenges associated with KT.

### 3.1 Urinary tract infections

UTIs are the most prevalent infections among KT recipients, with incidence rates ranging from 7% to 80% within the first-year post-transplantation (Coussement et al., 2018; Aydın et al., 2020; Coussement et al., 2020; Promsuwan et al., 2023; Szumilas et al., 2023). These infections are associated with significant complications, including sepsis, acute graft dysfunction, rejection, and even graft loss. Risk factors contributing to UTIs in this population include prolonged use of bladder catheters, immunosuppressive therapy, and the development of new-onset diabetes mellitus post-transplantation (Brennan et al., 2006; Cruz et al., 2018; Gerges-Knafl et al., 2020; Hsiao et al., 2021). UTIs in KT recipients can be categorized into asymptomatic bacteriuria, uncomplicated UTIs, complicated UTIs, and recurrent UTIs (Agrawal et al., 2022).

The predominant pathogens responsible for UTIs in KT recipients are Gram-negative bacteria, particularly *Escherichia coli*, *Enterococcus faecalis* (Abo Basha et al., 2019). Traditional diagnostic methods, such as urine culture, are considered the gold standard for identifying these pathogens. However, urine cultures can be time-consuming and may yield inaccurate results, especially if patients are undergoing antibiotic treatment (Janes et al., 2022; Kafi et al., 2022). Additionally, the emergence of drug-resistant strains, including extended-spectrum beta-lactamase (ESBL)-producing Gram-negative bacteria and carbapenem-resistant *Enterobacteriaceae*, poses significant challenges in the management of UTIs in KT recipients (Agrawal et al., 2022). Prophylactic antibiotic use in this population has shown limited efficacy and carries the risk of promoting resistant microorganisms (Wu et al., 2020). Studies have demonstrated that mNGS can detect a rich and diverse array of pathogens, with a significantly higher positive rate compared to traditional urine cultures. For instance, mNGS has shown an extraordinary positive detection rate in certain studies, surpassing the lower rates observed with conventional culture methods. Moreover, mNGS has proven effective in identifying viral, fungal, and mixed infections, which are often missed by standard diagnostic techniques. This comprehensive detection capability facilitates timely and targeted therapeutic interventions, thereby enhancing patient outcomes (Duan et al., 2022).

### 3.2 Pulmonary infections

Pulmonary infections are a leading cause of infection-related mortality in KT recipients (Huang et al., 2024). The spectrum of pathogens responsible for these infections is diverse and varies by region, including bacteria such as *Streptococcus pneumoniae*, *Escherichia coli*, *Klebsiella species*, *Pseudomonas aeruginosa*, and *Mycobacterium tuberculosis*, as well as viruses like *cytomegalovirus* (CMV) and BK virus (BKV) (Ahmad et al., 2020; Mangalgi et al., 2021; Qian et al., 2021; Bharati et al., 2023; Meira de Faria et al., 2023).

Conventional methods involve identifying potential pathogens using initial lab tests, imaging results, and exposure history, then conducting a thorough targeted assessment and treatment. Broad-spectrum empiric antibiotic therapy is considered for moderate to severe cases. Research has indicated that the mNGS technique is more sensitive in identifying pathogens in samples from transbronchoscopic lung biopsy (TBLB), bronchoalveolar lavage fluid (BALF), and bronchial needle brush (BB) compared to traditional culture methods (Dong et al., 2023). When diagnosing infectious pneumonia, mNGS of bronchoalveolar lavage samples provide more comprehensive results than transbronchial lung biopsy (Dong et al., 2023). MNGS can be more efficient in searching for pathogens in lung infections after KT, providing precise treatment, reducing costs, and improving cure rates, which is worthy of widespread application (Lian et al., 2024).

### 3.3 Bloodstream infections

BSIs are a significant concern in KT recipients, particularly within the first-year post-transplantation, with an incidence rate of approximately 10% (He et al., 2024). The primary pathogens involved are Gram-negative bacteria, notably *Escherichia coli, Klebsiella pneumoniae*, and *Pseudomonas aeruginosa* (Eviatar et al., 2022). In kidney transplant recipients, the primary sources of bloodstream infections are the urinary tract and access points for dialysis or central venous catheters. Approximately 35% of Enterobacteriaceae bacteria in this population are known to produce extended beta-lactamases (ESBL), which can hinder accurate diagnosis (Eviatar et al., 2022; Bharati et al., 2023). Traditional blood culture methods, while considered the gold standard, often require extended time to yield results and may fail to detect fastidious or non-culturable organisms, leading to delays in appropriate treatment. mNGS has emerged as a powerful diagnostic tool in this context. By sequencing all nucleic acids present in a blood sample, mNGS can rapidly identify a broad spectrum of pathogens, including bacteria, viruses, fungi, and parasites, without the need for prior knowledge of the causative agent. This comprehensive approach enables timely initiation of targeted antimicrobial therapy, which is crucial for reducing morbidity and mortality associated with BSIs in KT recipients. Moreover, mNGS can detect antimicrobial resistance genes, providing valuable information for optimizing treatment strategies (Tian et al., 2022).

### 3.4 Tuberculosis

Tuberculosis remains a leading cause of morbidity and mortality worldwide, and KT recipients are at an increased risk due to immunosuppressive therapy. The incidence of TB in KT recipients is significantly higher than in the general population, with rates reported to be 20-50 times greater (Vargas Barahona et al., 2022). TB in this population can result from reactivation of latent infection, donor-derived transmission, or new exposure post-transplantation (Krishnamoorthy et al., 2019). The clinical presentation is often atypical, and there is a higher likelihood of extrapulmonary or disseminated disease, complicating diagnosis and management (Zou et al., 2022).

Traditional diagnostic methods, such as the tuberculin skin test (TST) and interferon-gamma release assays (IGRAs), have limitations in immunocompromised patients, often yielding false-negative results (Li et al., 2022). Microbiological confirmation through culture is time-consuming and may delay treatment initiation. Moreover, certain anti-TB medications, like rifampicin, can interact with immunosuppressive drugs, necessitating careful management to prevent graft rejection (Hamon et al., 2023). mNGS offers a rapid and sensitive alternative for TB diagnosis in KT recipients. By detecting *Mycobacterium tuberculosis* DNA directly from clinical samples such as blood, sputum, or bronchoalveolar lavage fluid, mNGS facilitates early diagnosis, even in cases with atypical presentations or extrapulmonary involvement. Studies have reported that mNGS can identify TB infections with high sensitivity and specificity, enabling prompt initiation of appropriate therapy (Duan et al., 2021).

In conclusion, mNGS represents a significant advancement in the diagnosis of infections in KT recipients. Its ability to rapidly and accurately identify a wide range of pathogens, including those that are difficult to detect using conventional methods, makes it a valuable tool in the management of post-transplant infections. By facilitating early and precise pathogen identification, mNGS can guide targeted therapies, reduce the emergence of drug-resistant strains, and ultimately improve patient outcomes.

## 4 The role of mNGS in detecting various pathogens post-KT

KT recipients are particularly susceptible to infections due to immunosuppressive therapy, which can lead to severe complications (Sugi et al., 2019; Aydın et al., 2020; Xin et al., 2020; Dharia et al., 2022; Pilmis et al., 2023). Conventional diagnostic methods often fall short in promptly and accurately identifying the causative pathogens. mNGS has emerged as a powerful tool in this context, offering comprehensive pathogen detection across various infection types (Han et al., 2019; Liu et al., 2022; Chen et al., 2024) (**Table 2**).

**Table 2:** The advantages of mNGS in the detection of various types of pathogens in post-KT recipients.

Pathogen	Name of pathogen	Morbidity	Common symptom	Conventional methods	mNGS	References
Bacteria	G^-^ bacteria	Most often	Early hospital-acquired infections and urinary tract infections	Long incubation time and poor detection performance	Non-invasive, fast	(Li et al., 2022)
	G^+^ bacteria					
	TB	20-50 times higher than the general population	Unexplained persistent fever and unusual clinical presentation	False-negative results exist for PPD, IGRA	8/12 patients diagnosed with TB infection by NGS	(Zou et al., 2022)
	drug-resistant bacteria	Low morbidity	Difficult to diagnose and cure	All negative	Information for diagnosing drug resistance, virulence factors, and genomic variation.	(Liu et al., 2022)
Virus	CMV	8.8%-63.2%,	Acute transplant kidney injury, hematuria, fever, and dysuria	Low sensitivity of viral culture, long culture cycles, low sensitivity of serologic false negatives.	Viral nucleic acid detection sensitivity of 0.96, with a detection rate of 66% for mixed viruses	(Silva Junior et al., 2023)
	BKV	1%-10%	Loss of function of the transplanted kidney			(Wen et al., 2022)
	EBV	20% present within one year of transplantation	PTLD is the leading cause of cancer deaths in SOTR			(Bajda et al., 2020)
Fungus	PJP	The most common opportunistic fungal infections	Susceptible to the respiratory tract with multiple infections leading to death, often in combination with CMV	Cannot be cultured *in vitro*, BDG lacks specificity	The diagnostic sensitivity of mNGS for PJP was higher than that of GMS and BDG (100% vs. 15% and 74.5%, p < 0.001)	(Chen et al., 2020)
	IPA	Highest mortality rate	Lack of specificity in clinical presentation	G test, GM test low sensitivity, poor specificity	Confirmation of diagnosis and differentiation of Aspergillus species by mNGS	(Ma et al., 2022)
	Candida	46%-59.3%	Lack of specificity in clinical presentation	Low specificity of blood cultures, G tests	mNGS confirms the diagnosis	(Diba et al., 2018)
Rare pathogens	Corynebacterium striatum	23.3%	Heart Failure, Valve Superfluous Formation	Clinical microbiology results indicate infection, but no pathogens were cultured from specimens.	mNGS confirms the diagnosis	(Zheng et al., 2022)
	Talaromyces marneffei	30%	Lack of specificity in clinical presentation		mNGS confirms the diagnosis	(Xu et al., 2023)
	CVV	1%-19%	Manifests as meningitis		mNGS sensitivity was 86.1%, specificity was 97.9	(Al-Heeti et al., 2023)
	Rhodococcus equinus Equi	20-25%	Lack of specificity in clinical presentation		mNGS confirms the diagnosis	(Liang et al., 2024)

KT, kidney transplantation; mNGS, metagenomic next-generation sequencing; TB, Tuberculosis; CMV, *Cytomegalovirus*; BKV, *Cytomegalovirus*; EBV, *Epstein-Barr virus*; BKV, *BK virus*; PJP, *Pneumocystis jiroveci pneumonia*; IPA, *Invasive pulmonary aspergillosis*; CVV, *Cache valley virus*; BDG, beta-D-glucan test; PPD, purified protein derivative test; IGRA, interferon-gamma release assays; G test, 1,3-β-D-glucan test.

### 4.1 Frequent bacterial infections following KT

Bacterial infections are prevalent in the early stages following KT, with pathogens such as *Escherichia coli, Streptococcus pneumoniae, Klebsiella, Pseudomonas aeruginosa*, and *Enterococci* being common culprits (Bharati et al., 2023; Liu et al., 2024). Mixed bacterial infections are also frequent, and traditional culture methods often struggle to identify them effectively. Studies have demonstrated that mNGS significantly outperforms conventional methods in detecting mixed infections, with detection rates as high as 48.9% compared to 4.3% for traditional techniques (Zheng et al., 2022; Zhang et al., 2023). Additionally, mNGS can rapidly identify drug-resistant genes without the need for isolating resistant strains, thereby guiding the rational use of antimicrobial agents (Hao et al., 2023).

### 4.2 Common viral infections after KT

Viral infections, including those caused by *Cytomegalovirus* (*CMV*), *BK virus* (*BKV*), and *Epstein-Barr virus* (*EBV*), are significant contributors to morbidity and mortality post-transplantation (Savassi-Ribas et al., 2019; Nowak et al., 2021; Agrawal et al., 2022). CMV, a herpesvirus, is a common opportunistic infection that significantly affects kidney transplant outcomes, with prevalence rates among recipients ranging from 8.8% to 63.2% (Nowak et al., 2021; Silva Junior et al., 2023). CMV is strongly linked to complications such as pneumonia, hepatitis, uveitis, and acute or chronic rejection following transplantation. BKV infects 1–10% of kidney transplant recipients (Myint et al., 2021). While most individuals acquire BKV during childhood, with 80–90% of adults carrying the virus latently in renal tubular and urinary tract epithelial cells (Burek Kamenaric et al., 2020), immunosuppression can trigger a progression to BK virus-associated nephropathy (BK-VAN). In the U.S., BK-VAN affects 5–10% of kidney transplant recipients, with 50–80% of these cases resulting in graft failure (Srivastava et al., 2020; Kotla et al., 2021). EBV, another herpesvirus affecting 90% of adults, can lead to severe complications when reactivated, including post-transplant lymphoproliferative disorder (PTLD). PTLD accounts for 21% of cancers in transplant recipients and is a leading cause of cancer-related mortality in this population (Portuguese et al., 2023; Szumilas et al., 2023). Traditional methods for detecting viral infections post-transplant include molecular assays, antigenemia testing, histopathology, viral culture, and serological testing (Shirley et al., 2023). While viral culture is highly specific, its low sensitivity and lengthy turnaround time limit its clinical utility (Cui et al., 2023). Serological tests often fail in early-stage infections due to insufficient antibody levels, resulting in false negatives and low sensitivity (Nagarajah et al., 2020; Roubalová et al., 2020). PCR technology, especially quantitative real-time PCR (qPCR), has become the gold standard for diagnosing and monitoring viral infections due to its sensitivity and reliability (Peinetti et al., 2021). However, as a targeted method, PCR requires prior knowledge of the pathogen and is prone to false negatives, posing limitations in certain clinical scenarios.

Our research demonstrates that mNGS surpasses traditional tests in sensitivity for detecting postoperative lung infections in KT patients and aids in identifying viral infections. For suspected drug-resistant viral infections, mNGS is recommended to assess genotypic drug resistance (Kleiboeker, 2023). During the COVID-19 pandemic, mNGS played a vital role in diagnosing viral infections in kidney transplant recipients, detecting 15 viral nucleic acids with a sensitivity of 0.96 and identifying a wide array of viruses, including rare ones (Tian et al., 2022). Additionally, mNGS demonstrated a 66% detection rate for mixed viral infections (Tian et al., 2022). Prompt antiviral therapy guided by mNGS results can effectively control infections, reduce mortality, and minimize complications in transplant recipients.

### 4.3 Common fungal infections after KT

Fungal infections are a significant concern in KT recipients, with *Pneumocystis jiroveci pneumonia* (*PJP*), *invasive pulmonary aspergillosis* (*IPA*), and *candidiasis* being the most common types. *PJP*, caused by *Pneumocystis jiroveci*, often presents acutely or subacutely and is associated with increased graft failure and mortality (Zhang et al., 2019; Chen et al., 2020; Zhu et al., 2023). Traditional diagnostic methods, including microscopic examination, staining, and serum beta-D-glucan (BDG) testing, have low sensitivity and specificity, while obtaining respiratory specimens is challenging (Le Gal et al., 2019). mNGS offers superior sensitivity, enabling early detection of Pneumocystis jiroveci in blood or sputum and identifying mixed infections, with CMV being the most frequent co-pathogen (Zhang et al., 2021). mNGS significantly outperforms conventional tests like GMS and BDG in diagnostic accuracy, improving treatment outcomes (Wang et al., 2022).

Similarly, IPA, caused by Aspergillus species, remains a leading cause of mortality despite advancements in diagnostic techniques such as imaging, BDG, galactomannan assays, and fungal cultures, which often lack precision and are limited by invasive sampling requirements (Ma et al., 2022; Shi et al., 2023). mNGS provides a non-invasive, highly sensitive alternative, detecting various Aspergillus strains, including Aspergillus fumigatus and Aspergillus flavus, even in culture-negative cases (Zhang et al., 2021). It also identifies co-infections to guide antifungal therapy (Ma et al., 2022). For invasive candidiasis, traditionally diagnosed through blood cultures with low sensitivity, mNGS has demonstrated superior diagnostic accuracy, particularly in patients with underlying conditions or severe pneumonia (Huseynov et al., 2021; Atiencia-Carrera et al., 2022; Shi et al., 2023; Thomsen et al., 2024). By identifying Candida species and guiding targeted treatment strategies, mNGS significantly improves clinical outcomes for these high-risk patients.

Overall, mNGS is a transformative diagnostic tool, offering enhanced sensitivity and specificity across a range of fungal infections in KTR, enabling timely and effective interventions (Chen et al., 2020; Zhang et al., 2021; Zhang et al., 2021; Ma et al., 2022; Wang et al., 2022; Huang et al., 2023; Shi et al., 2023; Thomsen et al., 2024).

### 4.4 Infections by rare pathogens

mNGS demonstrates significant advantages in diagnosing rare infections such as *Streptococcus endocarditis*, *Toxoplasma marneffei*, *Cache Valley virus (CVV)*, *Rhodococcus equi*, and *Algeria Bacillus* (Zheng et al., 2022; Al-Heeti et al., 2023; Xu et al., 2023; Liang et al., 2024). It is particularly recommended when clinical suspicion of infection exists, but pathogens cannot be cultured from specimens. Unlike traditional methods, mNGS detects a broad range of pathogens simultaneously without requiring prior targeting (Liang et al., 2024). With superior accuracy and sensitivity, mNGS surpasses conventional techniques in pathogen detection, aiding clinicians in making timely and precise diagnoses (Zhang et al., 2023). It effectively identifies pathogens, including those transmitted through unconventional methods, and excels in diagnosing mixed infections (Weiqin et al., 2023). The technology offers faster detection, broader pathogen coverage, and greater clinical utility compared to traditional microbial culture. As sequencing technologies advance and costs decrease, mNGS is becoming increasingly integrated into clinical practice.

In summary, mNGS represents a significant advancement in the detection and management of diverse infections in KT recipients, offering rapid, comprehensive, and accurate pathogen identification that informs targeted therapeutic interventions.

## 5 Limitations and potential solutions of mNGS in post-KT infection diagnosis

While mNGS has demonstrated significant potential in diagnosing infections following KT, several limitations hinder its widespread clinical application (Zhong et al., 2021). One primary challenge is the high cost associated with mNGS, encompassing expenses for sequencing reagents, extraction, library preparation, and computational analysis. These costs often surpass those of traditional diagnostic methods, making routine use in clinical settings economically unfeasible (Zhong et al., 2021). Another significant limitation is the complexity of data interpretation. mNGS generates vast amounts of sequencing data, which can be challenging to analyze accurately. The presence of host DNA and commensal microorganisms can complicate the identification of pathogenic organisms, leading to potential misinterpretation of results. This complexity necessitates advanced bioinformatics tools and expertise, which may not be readily available in all clinical laboratories (Li et al., 2022; Li et al., 2023; Lu et al., 2023; Chen et al., 2024; Kan et al., 2024). Sensitivity and specificity issues also pose challenges. The detection of low-abundance pathogens can be difficult due to the overwhelming presence of host DNA, potentially leading to false negatives. Conversely, contamination or the presence of non-pathogenic microorganisms can result in false positives, complicating clinical decision-making (Han et al., 2019; Wang et al., 2020; Duan et al., 2022; Liu et al., 2022; Chen et al., 2024). Additionally, the lack of standardized protocols and bioinformatics pipelines hinders reproducibility across studies, and regulatory hurdles impede the integration of mNGS into routine clinical workflows (Ghosh et al., 2018; Hu et al., 2021).

To address these challenges, several strategies can be implemented. Reducing costs through technological advancements and streamlined workflows can improve the feasibility of mNGS for routine diagnostics. Developing standardized protocols and rigorous quality control measures can enhance data accuracy and reliability. Advanced bioinformatics pipelines are essential for effectively filtering out host DNA and distinguishing between pathogenic and non-pathogenic microorganisms, ensuring more precise data interpretation. Integrating mNGS with traditional diagnostic methods may provide a comprehensive approach, leveraging the strengths of both techniques to improve overall diagnostic accuracy. Hybrid sequencing approaches that combine short- and long-read technologies are also emerging as promising solutions, offering a balance between accuracy, cost, and read length.

In conclusion, while mNGS holds promise for diagnosing infections in KT recipients, addressing its current limitations is crucial for its effective integration into clinical practice. Ongoing research and technological advancements are essential to overcome these challenges and fully realize the potential of mNGS in improving patient outcomes.

## 6 Conclusion and future considerations

In conclusion, the application of mNGS has shown significant promise in revolutionizing the diagnosis and treatment of infections in KT recipients. This advanced technology enables the detection of a wide range of pathogens, including those that are difficult to identify using traditional microbiological methods. The ability to simultaneously identify bacteria, viruses, fungi, and parasites without the need for culture-based techniques provides a crucial advantage in the early diagnosis and management of post-transplant infections. Despite its potential, several challenges remain in the routine clinical adoption of mNGS for post-transplant infection diagnosis. These include the need for standardized protocols, cost-effectiveness considerations, and the integration of mNGS results into clinical decision-making. Furthermore, the interpretation of mNGS data can be complex, and it requires specialized expertise to distinguish between clinically significant pathogens and potential contaminants or colonizers.

Future research should focus on addressing these challenges. Efforts to streamline the data analysis process, improve the sensitivity and specificity of mNGS, and establish clear clinical guidelines for its use in post-KT infections will be crucial for its broader implementation. Additionally, longitudinal studies are needed to evaluate the long-term impact of mNGS on patient outcomes, including graft survival and overall survival. As technology advances, it is expected that mNGS will become an integral part of the diagnostic arsenal, improving the accuracy and timeliness of infection management in kidney transplant recipients. Moreover, exploring the role of mNGS in detecting emerging pathogens, monitoring antimicrobial resistance patterns, and guiding personalized therapeutic strategies will enhance its value in clinical practice. Collaborative efforts between clinicians, microbiologists, and bioinformaticians will be essential to maximize the full potential of mNGS in improving the care of KT patients.
